# Thermal Proteome Profiling in Zebrafish Reveals Effects of Napabucasin on Retinoic Acid Metabolism

**DOI:** 10.1074/mcp.RA120.002273

**Published:** 2021-02-13

**Authors:** Niels M. Leijten, Petra Bakker, Herman P. Spaink, Jeroen den Hertog, Simone Lemeer

**Affiliations:** 1 Biomolecular Mass Spectrometry and Proteomics, Bijvoet Center for Biomolecular Research and Utrecht Institute of Pharmaceutical Sciences, Utrecht University, Utrecht, the Netherlands; 2 Hubrecht Institute, KNAW and University Medical Center Utrecht, Utrecht, the Netherlands; 3 Institute Biology Leiden, Leiden University, Leiden, the Netherlands

**Keywords:** thermal proteome profiling, zebrafish, STAT3, napabucasin, aldehyde dehydrogenases, retinoic acid, Aldhs, aldehyde dehydrogenases, dpf, days postfertilization, NPARC, nonparametric analysis of response curves, RA, retinoic acid, ROS, reactive oxygen species, TPP, thermal proteome profiling

## Abstract

Thermal proteome profiling (TPP) allows for the unbiased detection of drug–target protein engagements *in vivo*. Traditionally, 1 cell type is used for TPP studies, with the risk of missing important differentially expressed target proteins. The use of whole organisms would circumvent this problem. Zebrafish embryos are amenable to such an approach. Here, we used TPP on whole zebrafish embryo lysate to identify protein targets of napabucasin, a compound that may affect signal transducer and activator of transcription 3 (Stat3) signaling through an ill-understood mechanism. In zebrafish embryos, napabucasin induced developmental defects consistent with inhibition of Stat3 signaling. TPP profiling showed no distinct shift in Stat3 upon napabucasin treatment, but effects were detected on the oxidoreductase, Pora, which might explain effects on Stat3 signaling. Interestingly, thermal stability of several aldehyde dehydrogenases was affected. Moreover, napabucasin activated aldehyde dehydrogenase enzymatic activity *in vitro*. Aldehyde dehydrogenases have crucial roles in retinoic acid metabolism, and functionally, we validated napabucasin-mediated activation of the retinoic acid pathway in zebrafish *in vivo*. We conclude that TPP profiling in whole zebrafish embryo lysate is feasible and facilitates direct correlation of *in vivo* effects of small molecule drugs with their protein targets.

## Highlights

• An optimized protocol for thermal proteome profiling in zebrafish embryos.
• Napabucasin affects thermal stability of several aldehyde dehydrogenases.
• Activating ALDH activity *in vitro* and retinoic acid pathway in zebrafish *in vivo*.
• Provides direct correlation of *in vivo* effects drugs with their protein targets.

## Graphical Abstract

**Figure:**
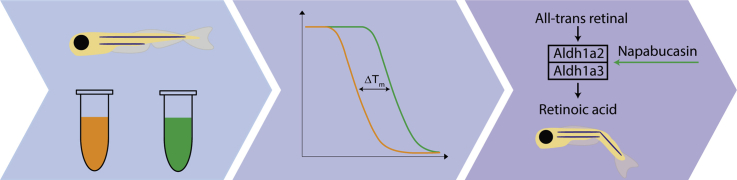

## In Brief

An optimized protocol for thermal proteome profiling in zebrafish embryo lysates has been developed. Using this protocol, it was shown that the Stat3 signaling inhibitor napabucasin stabilizes several aldehyde dehydrogenases. ALDH activity was activated by napabucasin *in vitro* and affected the retinoic acid pathway *in vivo*. These results show that thermal proteome profiling in whole zebrafish embryo lysate facilitates direct correlation of *in vivo* effects of small molecule drugs with their protein targets.

The thermal stability of proteins is influenced by their interaction with other (small) molecules (1). In recent years, large-scale studies using thermal proteome profiling (TPP) facilitated analysis of protein–protein, protein–metabolite, and protein–drug interactions, based on the changing thermostability of proteins (2). Especially in the field of drug discovery, the use of TPP facilitated the identification of new off-targets of clinically relevant drugs (3, 4, 5, 6).

Typically, a single cell type or—more recently—a single tissue (6) is being used as source of proteins. By using this approach, one may miss important drug targets because proteins are differentially expressed in cells and tissues (7), and the target proteins may not be expressed in the cells or tissues analyzed. Therefore, it would be interesting to perform TPP at an organism-wide scale to take all possible target proteins and their interactions in different tissues and cell types into account and obtain global insight into the mode of action of the drug–target interaction.

The zebrafish (*Danio rerio*) is an ideal model system, because its genome is very similar to the human genome (8) and orthologs of 84% of human disease–associated genes have been identified. Furthermore, external embryonic development is rapid with practically all organs developed at 2 days postfertilization (dpf). The embryos are transparent, allowing imaging of development and genetic manipulation of the zebrafish genome is relatively easy. Finally, the zebrafish model system is amenable to drug screens, in that treatment of developing zebrafish embryos with small molecule compounds may induce specific phenotypic changes (9). Because of these assets of the zebrafish, we hypothesized that zebrafish is an ideal model system for TPP on whole organisms.

To test the hypothesis that zebrafish embryos may be used to identify drug targets by TPP, we set out to identify protein targets of the small molecule napabucasin (BBI608). Napabucasin, a naphthoquinone, blocks stem cell activity in cancer cells and is being tested in multiple clinical trials as a mono or combination therapy (10). Napabucasin is thought to inhibit STAT3 signaling through a yet ill understood mechanism (11). In response to stimuli, cytosolic STAT3 is phosphorylated on Tyr705 by upstream tyrosine kinases (12), after which the phosphorylated protein homodimerizes because of an interaction between the phosphorylated tyrosine of one protein and the SH2 domain of another. The dimerized complex translocates to the nucleus, binds specific DNA fragments, and induces gene transcription. STAT3 activation has a central role in many biological processes. Transient STAT3 activation mediates wound healing and tissue integrity (13) and persistent STAT3 activation promotes tumor cell proliferation, survival, invasion, and immunosuppression (14). Treatment of cells with napabucasin leads to a decrease in the amount of phosphorylated STAT3 and hence, suppression of STAT3 signaling (15, 16, 17).

**Fig. 1:**
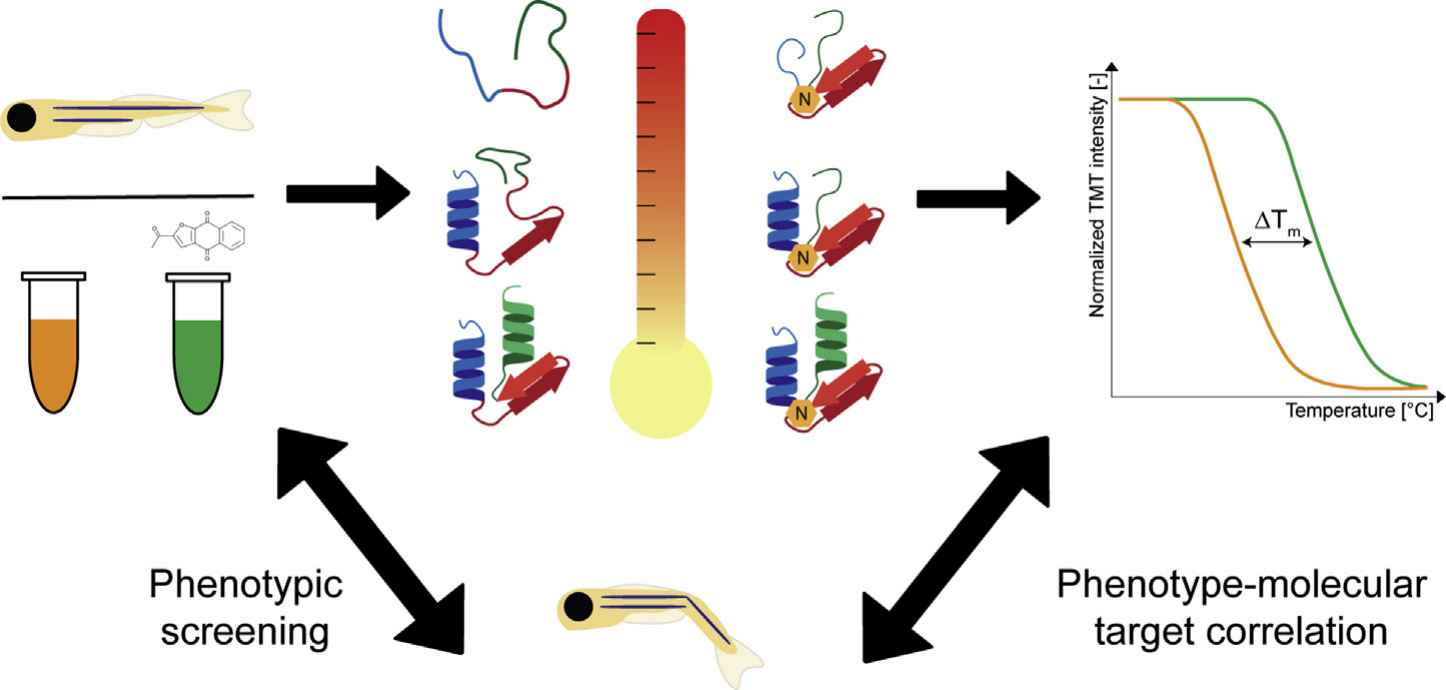
**Graphical representation of the workflow for zebrafish thermal proteome profiling.** Zebrafish embryo lysates were treated with either DMSO (control) or napabucasin before being subjected to heat treatment. If the drug binds to a protein, the complex can be stabilized leading to an increase in melting point. By quantification of the remaining soluble protein amount, melting curves can be generated. Phenotypic screening of zebrafish embryos using the same drug was done in parallel, which allows for direct correlation of the biological effects to the molecular drug targets.

Here, we report TPP at an organism-wide scale for the first time (Fig. 1). Using 5dpf zebrafish embryo lysates, targets of napabucasin were identified. Whereas napabucasin induced similar developmental defects as Stat3 knockdown, Stat3 did not show a stabilization shift, suggesting that Stat3 is not a direct target. However, several members of the aldehyde dehydrogenase (Aldh) family showed a thermal shift. Napabucasin enhanced Aldh activity, and we demonstrate that at least part of the effect of napabucasin on zebrafish embryonic development may be explained by its effect on retinoic acid (RA) metabolism *via* activation of Aldhs.

## Experimental Procedures

### Animal Husbandry

Adult zebrafish were maintained, and embryos were collected following natural mating as previously described (18, 19). All procedures involving experimental animals were approved by the local animal experiments committee (Koninklijke Nederlandse Akademie van Weterschappen-Dierexperimenten commissie) and performed according to local guidelines and policies in compliance with national and European law.

### Zebrafish Embryo Assays

Zebrafish embryos were injected at the one-cell stage with antisense Stat3 morpholino as described before (20). Alternatively, embryos were treated with napabucasin (5–10 μM), RA (10^−10^–10^−8^ M), Cyp26 inhibitor R115866 (1–10 μM), or control (0.2% DMSO). The embryos were imaged at 28 hours postfertilization to determine the distance from the otic vesicle to the tip of the nose using Image J. Alternatively, the embryos were fixed at 10.5 hours postfertilization or 18 hours postfertilization for *in situ* hybridization using *ntl*-specific (21) or *krox20/myoD*-specific probes (22, 23), respectively. The embryos were imaged, and the length of the *ntl*-stained notochord was determined using Image J.

### Experimental Design and Statistical Rationale

A total of 560 *D. rerio* embryos were pooled and lysed. From this pooled lysate, four replicates were taken, which were either treated with vehicle (2 samples) or drug of interest (2 samples). Samples were divided in 10 aliquots until TMT labeling, subsequently pooled, and divided in 10 fractions by high pH fractionation. All these fractions were injected separately in the LC-MS/MS system, and the following raw files were processed by MaxQuant.

### Thermal Profiling, Digestion, TMT Labeling, and Fractionation

Zebrafish embryos (5 dpf) were pooled and snap frozen. Embryos were reconstituted in lysis buffer (0.4% NP-40 in PBS + complete mini EDTA free protease inhibitors [Roche]) and lysed using bead-beating (Digital Disruptor Genie, Scientific industries) with zirconium oxide beads (1 mm). Subsequently, lysates were subjected to sonication (10 cycles, 30 s on/off) (Diagenode). Lysates were centrifuged for 30 min at 14,000 rcf and 4 °C to remove insoluble debris, after which the supernatant was transferred to a new tube and used for thermal proteome profiling.

Thermal proteome profiling was performed as previously described (2). Briefly, protein concentration was determined by a BCA protein assay kit (Thermo Fisher Scientific). The concentration of the lysate was adjusted to 2 mg/ml and divided in control and treatment. For the pervanadate experiment, lysates were treated with either PBS (control) or 100 μM pervanadate (24) for 20 min at room temperature. For the napabucasin (Bio-connect) experiment, lysates were incubated for 20 min with either DMSO (control) or 50 μM napabucasin (treatment). After treatment, lysates were divided in ten 100 μl aliquots. Heat treatment was performed in a PCR cycler (T100 thermal cycler, Bio Rad) for 3 min, cooled down to 25 °C for 3 min and afterward placed on ice. The temperature range spanned from 34 to 64 degrees, with increments of 3.3 °C. Precipitates were removed by ultracentrifugation (Beckman Coulter) at 125,000 rcf for 1 h at 4 °C. A volume corresponding to 100 μg of protein at the lowest temperature point was taken for further processing. Samples were reduced and alkylated by incubation with respectively 10 and 40 mM of tris(2-carboxyethyl)phosphine and chloroacetamide for 15 min. Afterward, samples were subjected to methanol/chloroform precipitation. The supernatant was removed, and the protein pellet was air dried. The protein pellet was dissolved in 6 M urea in PBS, after which a second reduction/alkylation step was performed using 10 mM tris(2-carboxyethyl)phosphine and 40 mM chloroacetamide for 15 min on the shaker. Samples were predigested with 1:100 LysC (protein: protease ratio) (Wako) for 2 h at 37 °C on a shaker. After predigestion, samples were further diluted to 1.5 M urea using 50 mM TEAB buffer (pH 8.5). Trypsin (Sigma) was added to a 1:100 ratio, and samples were digested overnight at 37 °C on a shaker. Digestion was stopped by acidifying to pH 2 by adding formic acid. Samples were centrifuged at 20,000 rcf for 10 min at 4 °C before they were desalted using 1 cc SEPPAK SPE cartridges (Waters). Briefly, cartridges were washed three times with 1 ml acetonitrile followed by washing three times with 1 ml of 0.1 M acetic acid. Samples were loaded after which the flow through was passed through the cartridges again. Cartridges were washed three times with 1 ml 0.1 M acetic acid after which the peptides were eluted using three times 250 μl 0.1 M acetic acid/80% acetonitrile. Samples were dried in a Thermo Savant SPD SpeedVac (ThermoFisher Scientific).

TMT labeling was performed as previously described (25). Briefly, protein digests were dissolved in 40 μl of 50 mM HEPES (pH 8.5) and mixed for 10 min at 20 °C. TMT reagents were dissolved in 42 μl 100% anhydrous acetonitrile, after which 10 μl of this solution was added to the peptides. Labeling was performed for 1 h at 20 °C while shaking at 400 rpm. The reaction was stopped by adding hydroxylamine to a final concentration of 0.4% and incubating for 15 min at 20 °C and 400 rpm. Subsequently, samples were pooled and acidified to pH 2 using formic acid. Samples were desalted using 1 cc SEPPAK SPE cartridges as described before. After desalting samples were dried using speedvac. Sample pools were fractionated using high pH reverse-phase HPLC fractionation using a Kinetex 5u EVO C18 100A column (Phenomenex) on a HPLC 1200 system (Agilent) operating at a flow rate of 200 μl/min. Briefly, Dried pellet was reconstituted in 20 μl of buffer A (10 mM NH4OH, pH 10) and injected. Samples were first loaded on the column at a flow rate of 20 μl/min for 2 min. Peptides were eluted stepwise using the following gradient: 2 to 12% buffer B (10 mM NH4OH/90% acetonitrile, pH 10) in 6 min, 12 to 35% buffer B in 47 min, 35 to 55% buffer B in 7 min, 55 to 100% buffer B in 3 min, 0 to 100% buffer A in 9 min, and 100% buffer A for 31 min. A total gradient time of 105 min was used. Fractions corresponding to 1 min of gradient time were collected on a 1260 infinity fraction collector (Agilent). Only fractions eluting after 8 min were collected. These fractions were concatenated in 10 fractions. All the fractions were dried down using speedvac and stored at −80 °C until further use.

### LC-MS/MS Analysis

For the TPP analysis, peptides were dissolved in 10% formic acid and a volume corresponding to 2 μg of peptides was injected on a UHPLC 1290 system (Agilent) coupled to a Q exactive HF-X mass spectrometer (Thermo Fisher scientific). Peptides were trapped (Dr Maisch Reprosil C18, 3 μm, 2 cm × 100 μm) before being separated using an analytical column (Agilent Poroshell EC-C18, 2.7 μm, 50 cm × 75 μm). Trapping was performed for 5 min in buffer A (0.1% formic acid) at a flow rate of 0.005 ml/min. The following gradient was used for separation: 12 to 42% buffer B (80% acetonitrile +0.1% formic acid) in 95 min, 100% buffer B for 2 min, followed by 100% buffer A for 11 min. The flow was split to generate a final flow of 300 nl/min. The Q Exactive HF-X was operated in a data-dependent acquisition mode with positive ionization. Full MS spectra were acquired from 375 to 1500 m/z at 60,000 resolution, using an automatic gain control target value of 3 × 10^6^ charges and a maximum injection time of 20 ms. A maximum of 12 precursors were allowed to be fragmented. A dynamic exclusion of 18 s was used. MS2 fragmentation spectra were obtained with a fixed first mass of 120 m/z at 45,000 resolution, using an automatic gain control target of 1 × 10^5^ and a maximum injection time of 85 ms. Fragmentation was performed using HCD at a NCE of 32.

### Protein Identification and Quantification

To identify peptides and proteins, raw files were processed using MaxQuant (version 1.6.5.0) and the Andromeda search engine, using the full Trembl database for zebrafish (55,769 entries, downloaded 22–07–2019). The following parameters were used: digestion by trypsin/P with a maximum of two missed cleavages, carbamidomethylation of cysteine as a fixed modification, oxidation of methionine, and N-terminal acetylation were selected as variable modifications. TMT 10plex labeling was used for quantification. The mass tolerance of precursor ions was chosen as ±5 ppm, and the mass tolerance of MS/MS was chosen as ±20 ppm. Results were adjusted to 1% peptide to spectrum matches and 1% false discovery rate using a target-decoy approach using reverted protein sequences.

Melting points were determined as previously described (2). In the first step, the relative abundances of the TMT reporter ions compared with the lowest temperature point were calculated. The lowest temperature point was set to 1. The experiments were normalized using the TPP R script (2), and melting curves were fitted according to the chemical denaturation theory:

$$f\left(T\right)\;=\;\frac{1\;-\;plateau}{1+e^{-\left(\frac{a}{T}\;-\;b\right)}}\;+\;plateau$$

In this equation, T is the temperature, and a, b and plateau are constants. The melting point of a proteins is determined as the temperature where half of the protein has denatured: f (T) = 0.5. The generated melting curves were inspected for a change in melting behavior. All melting curves shown were generated in GraphPad prism (8.3.0).

The generated melting curves were checked for significant difference by use of the NPARC method developed by Childs *et al.* (26), where we determined curves to be different if *p* ≤ 0.01. Additionally, we applied two more filters: (i) the melt point differences between vehicles and controls must have the same sign and (ii) the difference between the melting points of vehicle and control must be bigger than the difference between melting points of both vehicles. The remaining hits were manually screened.

### Sequence Alignment

All pair wise sequence alignments were performed with the EMBOSS Needle algorithm (27).

### ALDH Activity Assay

ALDH activity colorimetric assay kit (Sigma-Aldrich) was performed according to manufacturer’s protocol. Briefly, HepG2 cells (30 million) were resuspended in ALDH assay buffer before being lysed by freeze/thaw cycling. The lysate was treated with 1% DMSO (control), 50/100 μM Napabucasin, or 20/50/100 μM ALDA-1 for 30 min before the colorimetric assay was started. Absorbance was measured at 450 nm on a Multiskan GO plate reader (Thermo Scientific), which was subsequently converted to the ALDH activity.

## Results

### Optimizing Thermal Proteome Profiling for Zebrafish Embryos

Zebrafish embryo development is well underway at 5 dpf, and most organs and tissues have formed, including cartilage. Accordingly, the original TPP protocol (2, 3) was adjusted to make it amenable to zebrafish. Lysis was performed by bead beating followed by sonication which was necessary to disrupt the embryo. The lysis buffer contained the mild detergent NP-40 to allow the generation of melting curves for membrane proteins (28). In this first attempt at organism-wide TPP, the workflow was performed on lysate to keep the complexity of analysis as low as possible. By way of a positive control, we anticipated that pervanadate, a strong oxidative agent, would have a global effect on the thermal stability of the proteome. The lysates were treated with either 100 μM pervanadate or PBS as negative control and incubated for 20 min at RT before they were subjected to heat treatment. Zebrafish normally live at 28 °C. Human cell lines that have been used to date for TPP grow at 37 °C. To avoid potential problems resulting from this temperature difference, a broad range of temperatures was tested for TPP on zebrafish lysates. A temperature range of 34 to 64 °C is optimal for the generation of melting curves of zebrafish embryo lysates (supplemental Fig. S1).

Our pilot study resulted in the identification of a total of 6592 proteins, of which 5159 had at least two unique peptides (supplemental Tables S1 and S2). Differential melting behavior was determined through the nonparametric analysis of response curves (NPARC) method (26). The global analysis of the melting behavior of proteins shows a small global increase in protein stability in the pervanadate treated samples (supplemental Fig. S2) and indicates an infliction point at around 50 °C where the majority of proteins start to precipitate. Distinct classes of proteins showed a shift, including multiple ATPases and proteins involved in the citric acid cycle and glycolysis (supplemental Fig. S2). All melting characteristics of the identified proteins can be found in supplemental Table S3. Taken together, our results showcase the validity of thermal proteome profiling on total zebrafish embryo lysates.

### Probing the Targets of Napabucasin by Thermal Proteome Profiling in Zebrafish Embryos

**Fig. 2:**
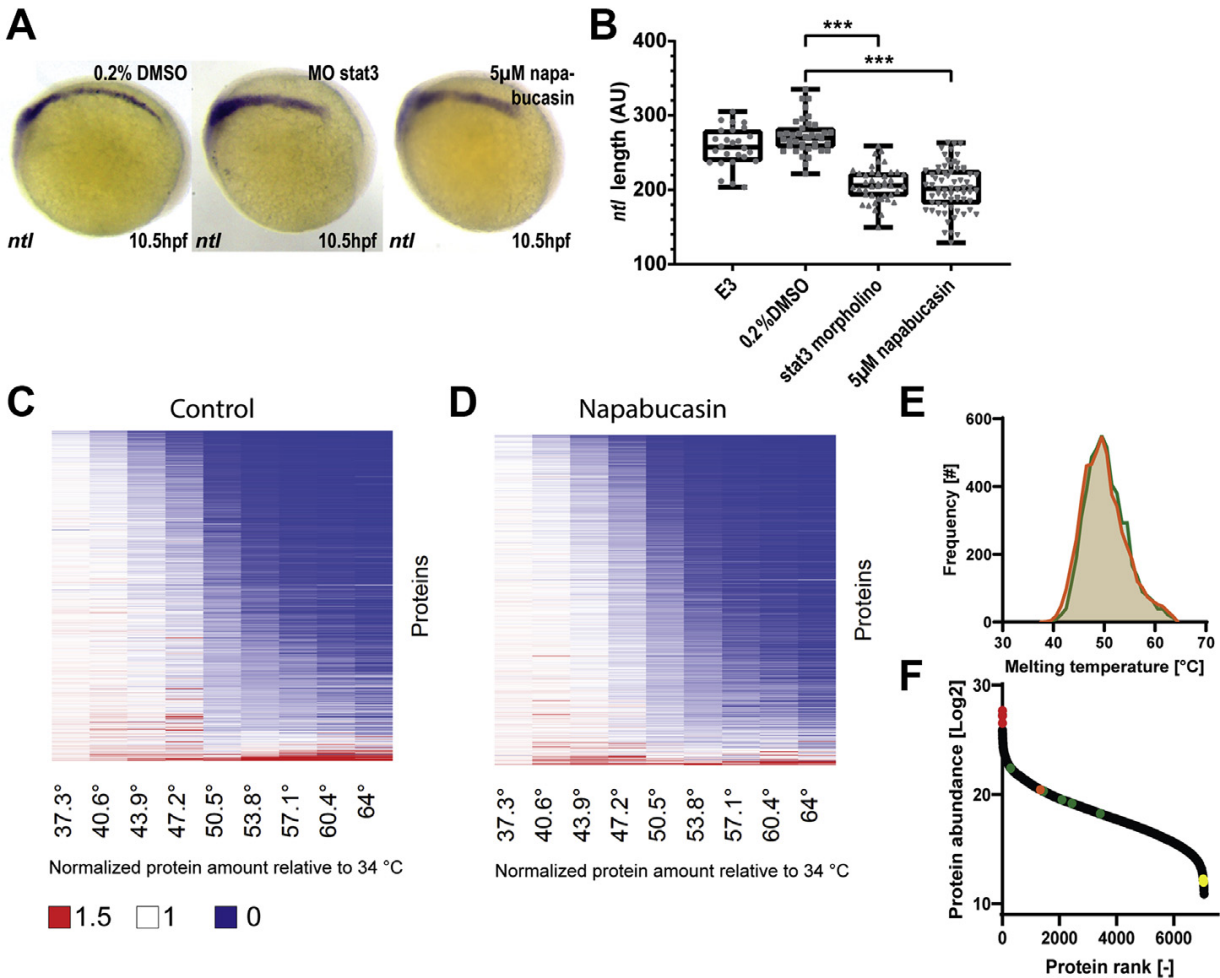
**Thermal proteome profiling of Napabucasin in zebrafish embryos**. Embryos were treated with napabucasin (5 μM) or solvent control (0.2% DMSO). Alternatively, antisense STAT3-specific morpholino (MO stat3) was microinjected at the 1-cell stage. *A*, at 10.5 hpf, the embryos were fixed, and *in situ* hybridization was performed using a *ntl*-specific probe, which stains the notochord. *B*, the length of the notochord was determined in napabucasin treated and *stat3*-morpholino knockdown embryos compared with DMSO and nontreated E3 medium control. Significance was determined using a one-way ANOVA with Dunnetts multiple comparisons test, n = 26 to 66 embryos per condition; ∗∗∗*p* < 0.001. The global precipitation behavior of proteins in DMSO (*C*) or napabucasin treated (*D*) lysate is similar, in agreement with overall comparable melting points for proteins under these two conditions (*E*). The protocol captured a large dynamic range of proteins, ranging from high abundant proteins such as Vtg1, Ttnb, and Myhz1.2 (*red*) to low abundant proteins such as Sirt1, Cbx5 and Bbs7 (*yellow*). Additionally, Stat3 (*orange*) and all shifting Aldh proteins (*green*) are shown (*F*). hpf, hours postfertilization.

Next, we sought to probe the feasibility of using zebrafish TPP with a more selective inhibitor. Napabucasin has an effect on stemness of cancer cells and may act through inhibition of STAT3 signaling (11). Liu *et al.* (29) have shown that zebrafish embryos lacking functional Stat3 display a shortened notochord compared with wildtype embryos, which is caused by reduced cell proliferation and increased apoptosis. We tested napabucasin (5 μM) treatment on zebrafish embryos for phenotypic changes, using *ntl in situ* hybridization staining as read-out for a shortened notochord. As a control, morpholino-based antisense oligonucleotide knockdown of Stat3 was used, which induced a shortened notochord, comparable to the genetic knockout. Treatment with DMSO (solvent) was used as a negative control. Treatment with napabucasin induced significant shortening of the notochord, comparable to the effect of morpholino-mediated Stat3 knockdown (Fig. 2, *A*–*B*). These results suggest that napabucasin may have a direct effect on Stat3 signaling and thus affect zebrafish embryonic development.

A TPP experiment was performed to identify direct protein targets of napabucasin. Lysates from 5 dpf zebrafish embryos were treated with 50 μM napabucasin or DMSO as a control for 20 min, before heat treatment. Seven thousand six hundred forty-six proteins were identified, of which 6114 proteins with at least two unique peptides, making this the largest proteomics dataset for zebrafish till date (supplemental Tables S4 and S5). The experiment captured a large dynamic range of proteins: high abundant proteins such as the egg yolk protein vitellogenin (vtg1) and muscle components titin (ttnb) and myosin (myhz1.2) were detected (Fig. 2*F*). At the same time, low abundant proteins such as the protein deacetylase sirtuin 1 (Sirt1), heterochromatin component Cbx5, but also the ciliar protein Bbs7 are detected. This showcases the potential of detecting proteins with intensities spanning multiple orders of magnitude using this protocol. Differential melting behavior was determined through the NPARC method (26). Heat maps displaying the global profile show that overall thermal behavior between DMSO and napabucasin-treated samples was highly similar (Fig. 2, *C*–*D*). Comparison of the distribution of melting points indicates that only a small population of proteins showed a different melting behavior after drug treatment (Fig. 2*E*). All melting characteristics of the identified proteins can be found in supplemental Table S6. We selected multiple proteins as examples and found that the change in thermal stability of these proteins is distinct between napabucasin and pervanadate treatment (supplemental Fig. S3).

**Fig. 3:**
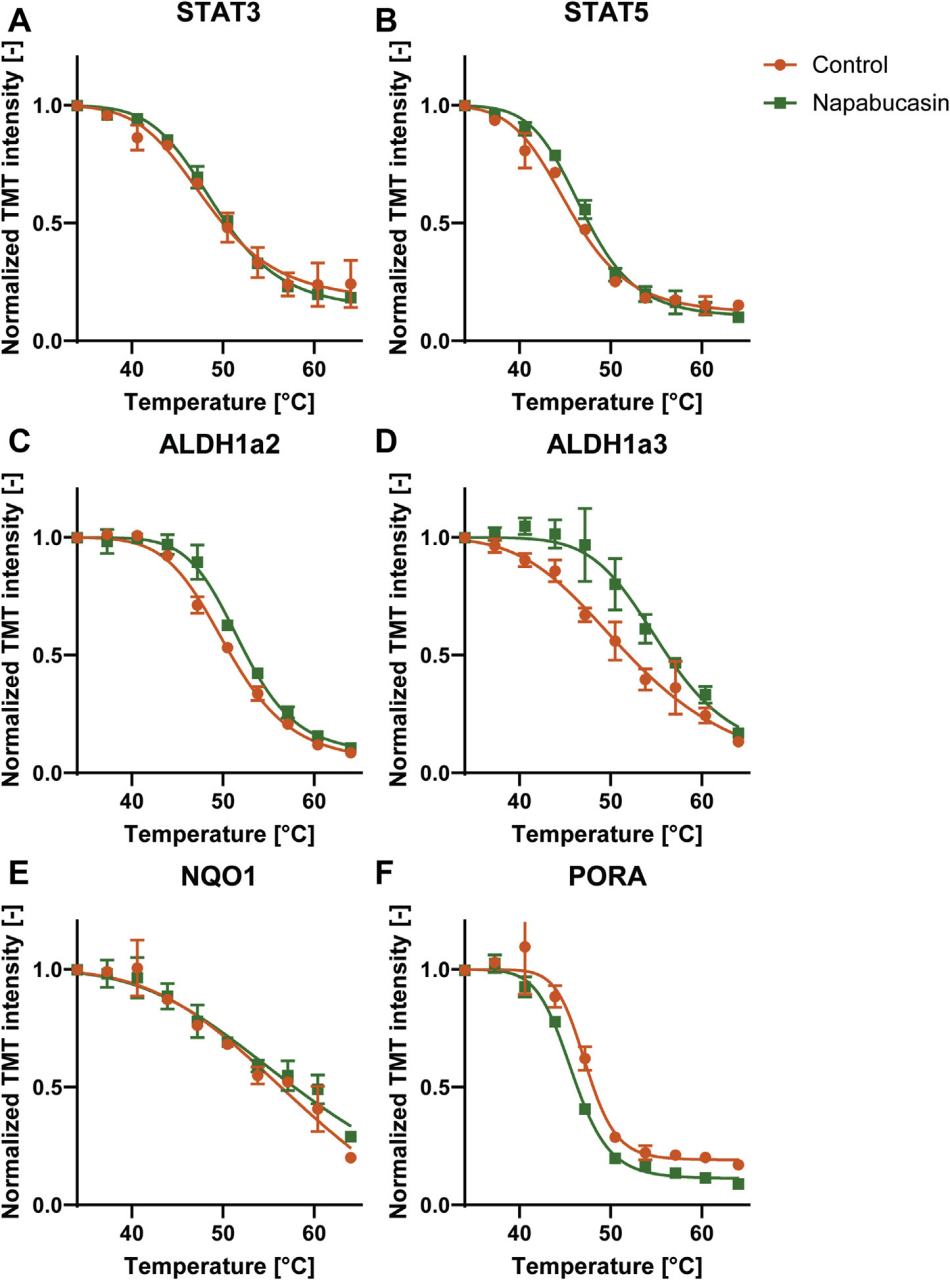
**TPP indicates differential effects of napabucasin treatment on selected proteins.** Stat33 (*A*) does not show any shift, whereas Stat5 (*B*) shows a small shift. However, a shift was detected in Aldh1a2 (*C*) and Aldh1a3 (*D*). The known interactor of napabucasin Nqo1 (*E*) shows no shift. Interestingly, another known interactor, Pora, (*F*) shows a shift indicating destabilization. Data points (n = 2 independent experiments) are shown as mean ± SEM, melting curve fitting was performed according to chemical denaturation theory.

No shift in Stat3 and only a minimal nonsignificant shift in Stat5 were observed (Fig. 3, *A*–*B*). Stat3 was readily detected with high abundance (Fig. 2*F*) and good sequence coverage (37.3%), indicating that the absence of shift is likely because of the absence of interaction between protein and drug. However, an interesting class of proteins showed a stabilizing effect because of napabucasin, the Aldhs (Fig. 3, *C*–*D*). These stabilized Aldhs are detected with high abundances, indicating high data quality (Fig. 2*F*). All Aldhs that were detected in this assay can be found in supplemental Fig. S4*A*. Interestingly, the stabilized Aldh proteins show a high degree of conservation with each other, whereas conservation with nonshifting Aldhs is limited (supplemental Fig. S4*B*). Aldh proteins play a role in converting endogenous and exogenous aldehydes to the corresponding carboxylic acids (30), and one of the most important *in vivo* substrates of Aldh enzymes is retinaldehyde, which is converted into RA, an essential morphogen in vertebrate development. Pora, an oxidoreductase, was also destabilized by napabucasin, whereas another oxidoreductase, Nqo1, did not shift (Fig. 3, *E*–*F*). It is noteworthy that napabucasin is a substrate of oxidoreductases (17). Altered thermal stability of Pora, but not Nqo1, may reflect direct interaction of napabucasin with Pora, but not Nqo1.

**Fig. 4:**
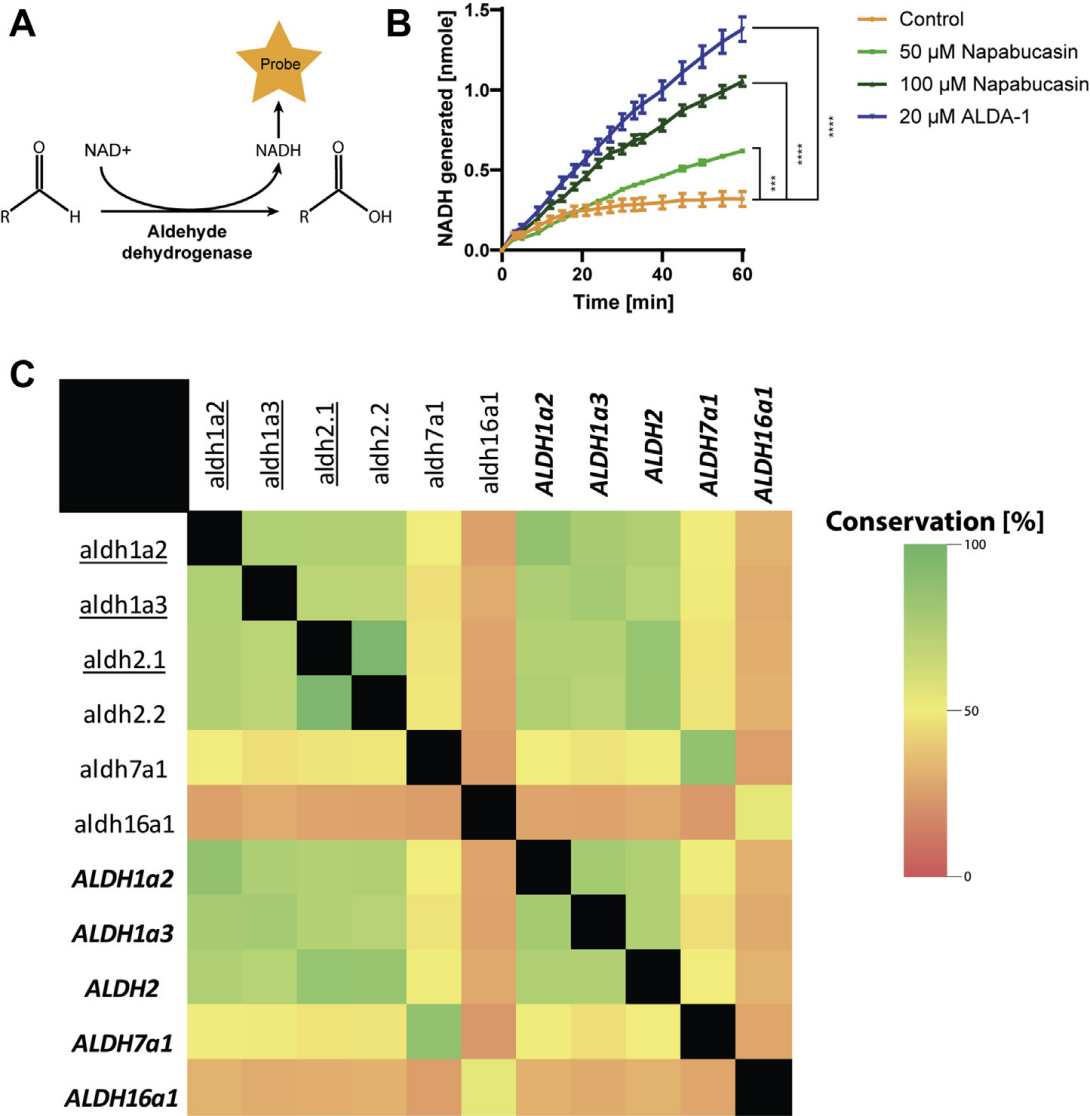
**Activation and conservation of human ALDHs.** The ALDH assay measures the conversion of NAD+ to NADH using a colorimetric probe (*A*). ALDH enzymatic activity was determined in lysates of HepG2 cells. Two concentrations of napabucasin were used (50 μM and 100 μM) as well as a known ALDH activator, ALDA-1 (20 μM). 1% DMSO was used as a control, and experiments were performed in triplicate. *p*∗∗∗ <0.001, *p*∗∗∗∗ <0.0001. Significance was determined by one-way ANOVA (*B*). The shifting (underlined) zebrafish aldh1a2, aldh1a3, and aldh2.1 show a large degree of conservation with their human counterparts (shown in *italic* and *bold*), but show no large conservation with other zebrafish or human Aldh enzymes (*C*). Aldhs, aldehyde dehydrogenases.

We investigated the interaction between napabucasin and ALDH proteins by analysis of human ALDH enzymatic activity. ALDH expression is especially high in liver cells, and therefore, we assessed the effect of napabucasin on ALDH in lysate of HepG2 human liver cells in a colorimetric assay. Surprisingly, napabucasin had a significant activating effect on ALDH enzymatic activity. As a control, we used the known ALDH2 activator ALDA-1 (31), which also showed elevated ALDH activity in HepG2 cell lysates (Fig. 4, *A*–*B*). Taken together, these data show that napabucasin interacts with human ALDHs, thereby activating their activity.

Sequence alignment of zebrafish and human ALDH proteins reveals high sequence conservation between zebrafish retinaldehyde converting Aldhs (*aldh1a2* and *aldh1a3*) and the human retinaldehyde converting ALDHs, providing a proper explanation for an interaction of napabucasin with human ALDHs (Fig. 4*C*). Additionally, the retinaldehyde converting Aldhs show a large conservation with human, acetaldehyde converting ALDH2, explaining the results of the colorimetric assay on HepG2 cells, which used acetaldehyde as substrate. It is noteworthy that the zebrafish Aldh2.1 also shows a stabilizing effect because of napabucasin, indicating an interaction; however, because of large conservation, only a single unique peptide for this protein could be detected. These results combined proof the use of our zebrafish model as a platform to study drug function in humans.

### Validation of the Interaction of Napabucasin With ALDHs by Using a Zebrafish Phenotypic Model

**Fig. 5:**
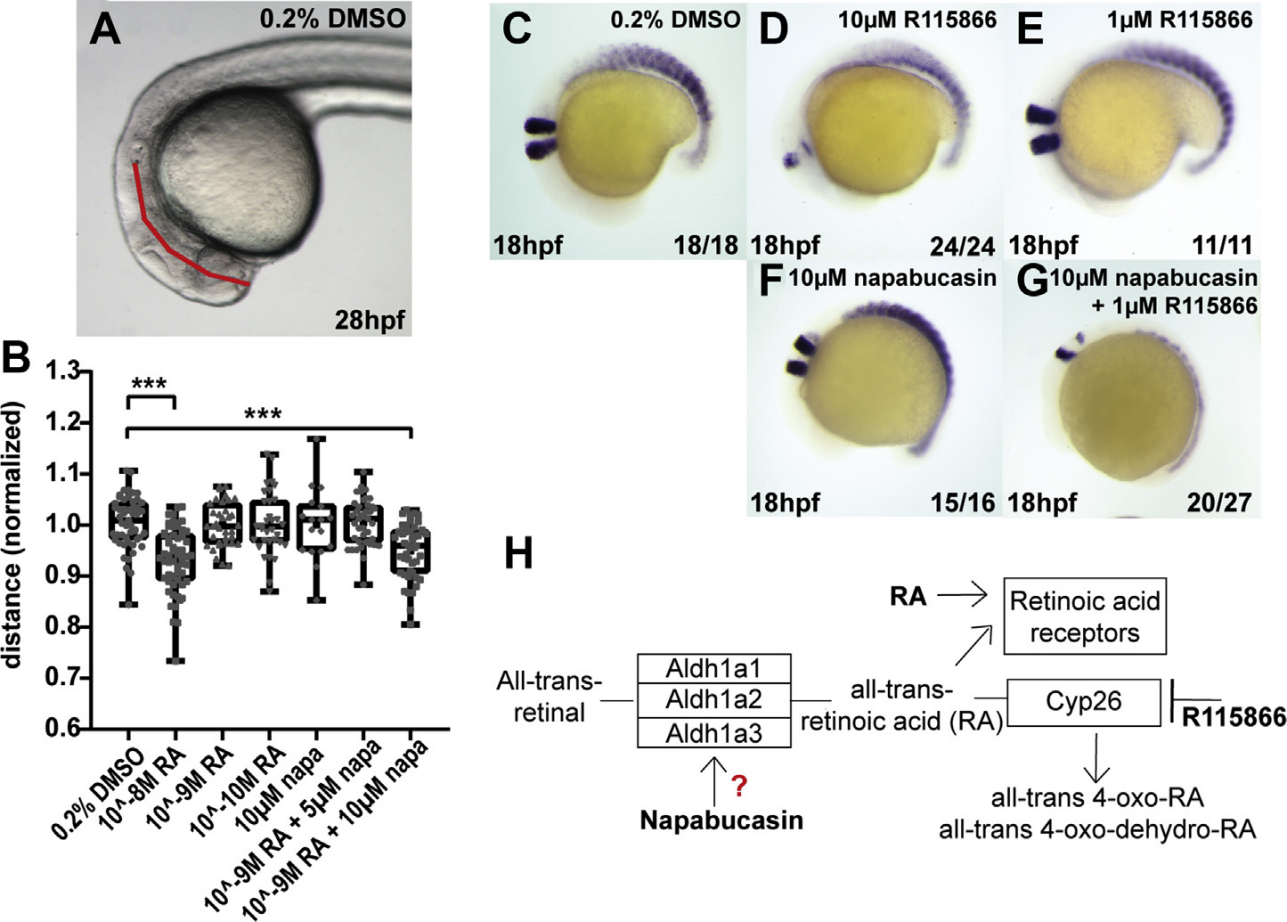
**Validation of the off-targets of napabucasin using a zebrafish phenotypic model.** The distance between the otolith and the tip of the nose was determined (indicated with a *red line*) in control (0.2% DMSO) RA treated (10^−8^, 10^−9^ or 10^−10^ M), napabucasin (10 μM) or combinations, as indicated (*A*–*B*). Significance was determined using a one-way ANOVA with Dunnetts multiple comparisons test. Individual dots represent individual embryos; minimally 26 and maximally 66 embryos were used per condition; ∗∗∗*p* < 0.001. Zebrafish embryos were treated with the Cyp26 inhibitor, R115866, with napabucasin or combinations. The embryos were fixed at 18 hpf, and *in situ* hybridization was performed using *krox20* and myod-specific probes, marking rhombomeres (3 and 5) and the somites, respectively (*C*–*G*). Schematic representation of RA metabolism and the role of Aldhs and Cyp26 in the process (*H*). Aldhs, aldehyde dehydrogenases; hpf, hours postfertilization; RA, retinoic acid.

The Aldh family of enzymes has a crucial role in RA metabolism *in vivo* (32). RA is an essential morphogen in vertebrate development and regulates cellular processes, embryo patterning, and organogenesis. Enhancing RA levels by exogenous addition of RA or by inhibition of RA catabolizing enzymes, such as Cyp26, results in severe developmental defects, most prominently in development and patterning of anterior neural structures. In zebrafish embryos, treatment with exogenous RA induces defects in hindbrain development, resulting in a significant reduction of the distance from the otic vesicle to the tip of the nose (33). Treatment with exogenous RA and inhibition of Cyp26 was reported to interfere with normal development of rhombomeres 3 and 5 (34, 35, 36). Because napabucasin enhanced Aldh enzymatic activity, we hypothesized that napabucasin treatment would induce similar developmental defects as treatment with exogenous RA and/or inhibition of Cyp26. Treatment with 10^−8^ M RA induced a significant reduction of the distance between the otic vesicle and the tip of the nose, whereas treatment with 10^−9^ M or 10^−10^ M did not (Fig. 5, *A*–*B*, supplemental Fig. S5). Napabucasin treatment (10 μM) by itself did not induce significant defects. Interestingly, combined treatment of embryos with 10 μM napabucasin and 10^−9^ M RA induced a significant reduction in the distance between the otic vesicle and the tip of the nose, suggesting that napabucasin and RA cooperate to induce these defects. Note that at these concentrations, napabucasin and RA by themselves did not induce developmental defects. Higher concentrations of napabucasin induced severe developmental defects, which precluded assessment of the distance between the otic vesicle and the tip of the nose.

Inhibition of Cyp26 with R115866 induced defects in the development of rhombomeres 3 and 5 in a dose-dependent manner, assessed by *krox20 in situ* hybridization (Fig. 5, *C*–*E*). The myoD-specific marker was included to mark the somites. Napabucasin (10 μM) by itself did not affect rhombomere development but co-treatment of embryos with 1 μM R115866, which did not affect rhombomere development by itself, did induce defects (Fig. 5, *F*–*G*), indicating that napabucasin and R115866 cooperate. Napabucasin enhanced Aldh enzymatic activity, resulting in enhanced RA production, and R115866 reduced Cyp26 activity, which also results in enhanced RA levels (Fig. 5*H*). Our results suggest that at least part of the *in vivo* function of napabucasin is mediated by elevation of RA levels in zebrafish embryos.

## Discussion

This study shows the applicability of zebrafish as model system for thermal proteome profiling on whole organisms. First, a proof of principle experiment was performed with the broad-range oxidative agent pervanadate. A part of the proteome shows a stabilizing effect because of pervanadate treatment, clearly indicating the possibility of measuring treatment induced stability changes. Focusing on specific proteins showed a shift in ATPases (supplemental Fig. S2). It has been reported that vanadate binds to ATPases in the catalytic site, which causes thermal stabilization (37). These results show that ligand-induced stabilization can be detected in zebrafish lysates on a proteome-wide scale.

The biggest advantage of using whole organisms compared with a single cell type is the increased diversity of proteins that can be investigated. In our proteomics data, multiple proteins from specific tissue types were found (38). Amylase (pancreas), glial fibrillary acidic protein (cerebral cortex), myosin binding protein C (heart muscle), and apolipoprotein A-II (liver) were found, amongst others. This indicates the possibility of screening all proteins of a whole organism using TPP, including tissue-specific proteins which would be missed if single cell types or tissues would have been used. It is evident that highly and broadly expressed proteins are more highly represented in whole organism lysates than lowly expressed proteins or proteins that are exclusively expressed in specific, low abundant cell types. Current mass spectrometry technology is instrumental in identifying low abundant proteins in a background of highly expressed proteins. In this respect, it is noteworthy that we identified the largest zebrafish proteomics dataset reported to date, containing 7646 unique proteins. We managed to identify proteins at different scales of magnitude, from the highly abundant vtg1 to low abundant proteins such as sirt1.

We used zebrafish embryo lysates to identify targets of napabucasin, a drug that reportedly affects STAT3 signaling. The melting curves of Stat3 did not indicate a thermal shift in response to napabucasin. This may suggest that there is no binding between the drug and Stat3. Recent literature suggests that the mode of action of napabucasin involves the oxidoreductases POR and NQO1, which generate reactive oxygen species (ROS) (17). Interestingly, we observed a destabilizing effect of napabucasin on the more highly conserved (71%) homolog of POR, Pora, than on the less conserved (49%) homolog of Nqo1, (Fig. 3, *E*–*F*). Hence, napabucasin may modulate Pora in zebrafish, resulting in increased ROS levels, leading to a decrease in phospho-Stat3 and Stat3 levels (39), which might explain the phenotypic similarity between napabucasin treatment and morpholino-mediated knockdown of Stat3 (Fig. 2*A*).

Multiple members of the Aldh family of proteins were stabilized upon napabucasin treatment. ALDHs play an important role in aldehyde metabolism, by catalyzing the oxidation of reactive aldehydes (40), thereby reducing the level of ROS. ALDH activity is also necessary for the generation of vital biomolecules, including RA and folate (41). Our data convincingly showed that napabucasin increased ALDH enzymatic activity *in vitro* (Fig. 4*B*). It is noteworthy that zebrafish Aldh1a2 and Aldh1a3 show a large degree of conservation with human ALDH2. This observation explains why the colorimetric assay, which uses the ALDH2 preferred acetaldehyde as substrate, shows an increase in human ALDH activity after napabucasin treatment. The effect of napabucasin on zebrafish development *in vivo* was consistent with Aldh activation, in that napabucasin treatment cooperated with suboptimal RA treatment and inhibition of RA catabolism (Fig. 5).

Napabucasin blocks stem cell activity in cancer cells and is being tested in multiple clinical trials as anticancer drug. It will be interesting to investigate in future experiments whether napabucasin-induced activation of ALDH activity and subsequent elevation of RA levels also has a role in the effect of napabucasin on stemness of cancer stem cells.

In this project we improved the traditional TPP workflow and applied it to whole zebrafish embryo lysates instead of only a single cell type or tissue. This allowed us to screen the proteome for targets of napabucasin. Multiple Aldh family proteins were identified as targets of napabucasin. Our data support the conclusion that developmental defects in napabucasin-treated zebrafish embryos may result from activated Aldh-mediated elevation of RA levels.

## Data Availability

The datasets reported in this article have been deposited in the ProteomeXchange Consortium *via* the PRIDE (42) partner repository PXD017418 (pervanadate) and PXD017419 (napabucasin). Mass-labeled MS/MS spectra were supplied to MS-Viewer (27) with search keys **lea6ryrs0n** (pervanadate) and **bbxsm2el6a** (napabucasin).

## Conflicts of Interest

The authors declare no competing interests.
