# Supplementary material for: Thermal Proteome Profiling in Zebrafish Reveals Effects of Napabucasin on Retinoic Acid Metabolism
Source: Mol Cell Proteomics. 2021 Feb 13;20:100033. doi: 10.1074/mcp.RA120.002273 (PMC7950114; doi:10.1074/mcp.RA120.002273)
Supplement: Supplemental Table S2 [file mmc3.pdf]

Supplementary figures for the paper: Thermal proteome profiling in zebrafish reveals effects of napabucasin on retinoic acid metabolism

Niels M. Leijten[1*], Petra Bakker[2,3*], Herman P. Spaink[3], Jeroen den Hertog[2,3#], Simone Lemeer[1#]

Table of contents:

Figure 1: SDS-page gel showing the optimal heat treatment for zebrafish embryos

Figure 2: TPP experiment on zebrafish embryos using pervandate

Figure 3: Comparison of melting behavior of proteins in both pervanadate ne napabucasin experiments

Figure 4: Coverage of all detected ALDH proteins and their conservation in zebrafish

Figure 5: Representative images for measurement of the distance between otolith and tip of the nose in zebrafish embryos.

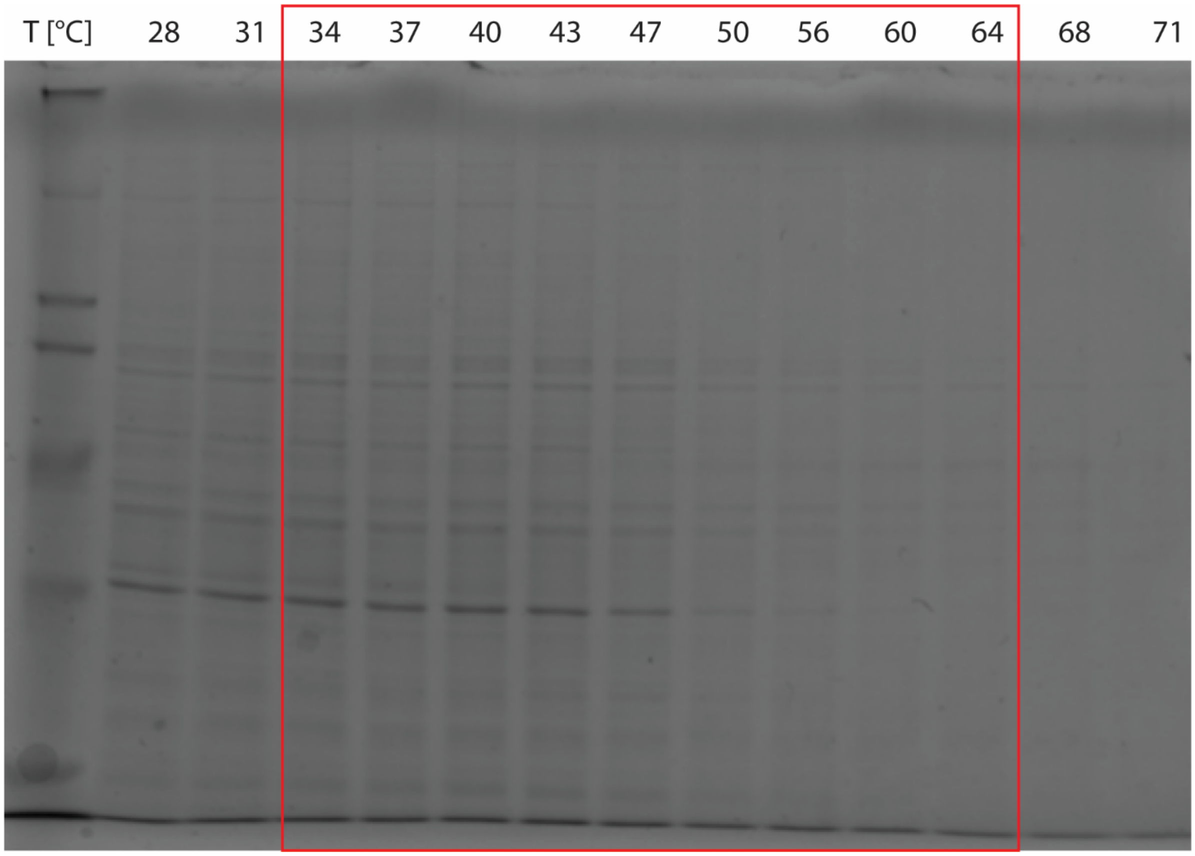

Supplementary Figure 1: SDS-page of zebrafish lysates heated to different temperatures. The temperature range of 34 – 64 °C is optimal for the generation of melting curves.

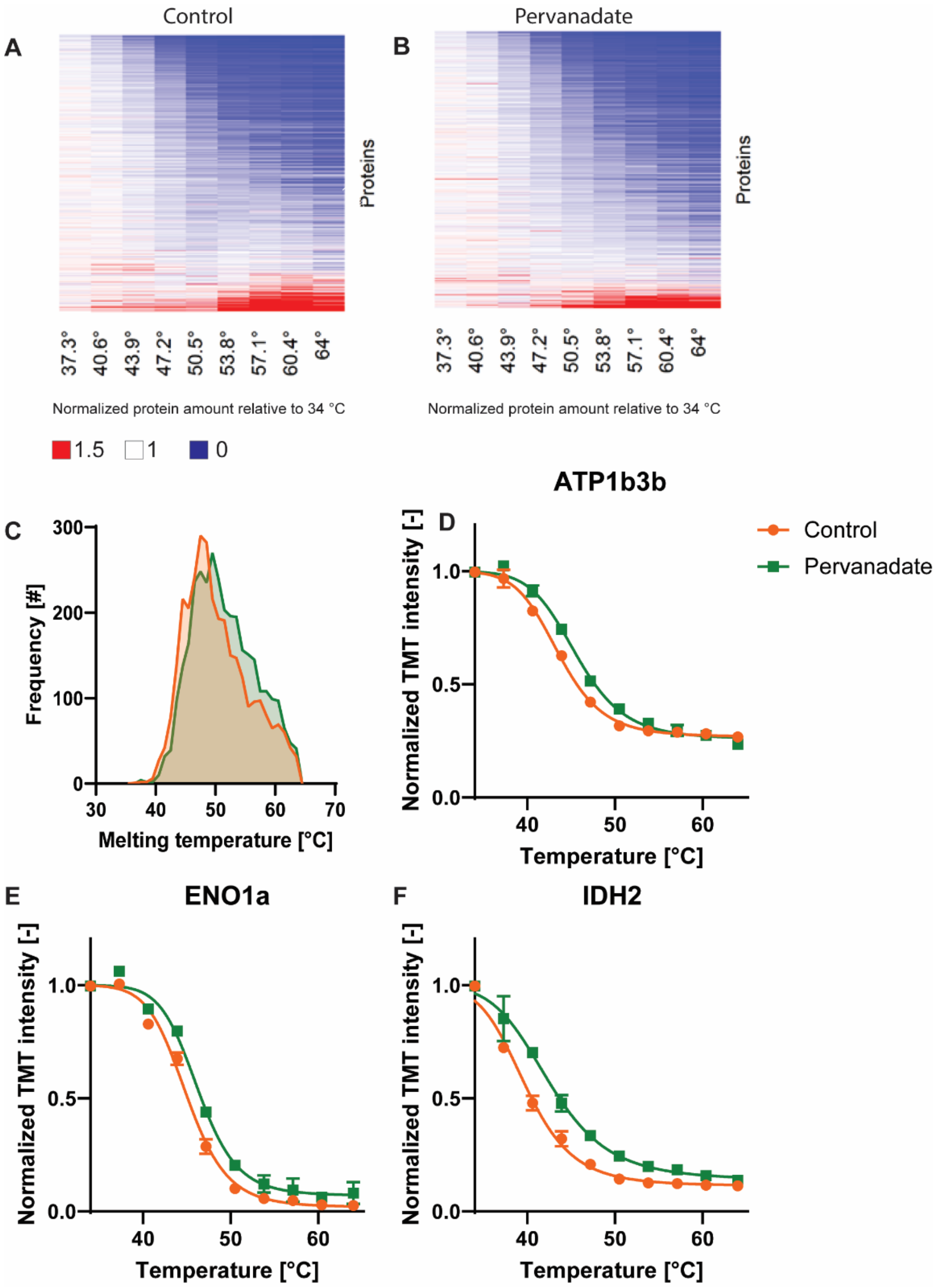

Supplementary Figure 2: TPP experiment using pervanadate. Heat maps of the precipitation behavior of control (A) and pervanadate treated (B) lysate shows that there is a global stabilization in the pervanadate treated samples, which is reflected in a global increase in melting points in pervanadate treated lysates (C). Some proteins have a shifted melting curve, such as ATPases (D), proteins involved in glycolysis such as enolase (E) and proteins involved in the citric acid cycle such as isocitrate dehydrogenase (F). Data points (n = 2 independent experiments) are shown as mean ± SEM, melting curve fitting was performed according to chemical denaturation theory.

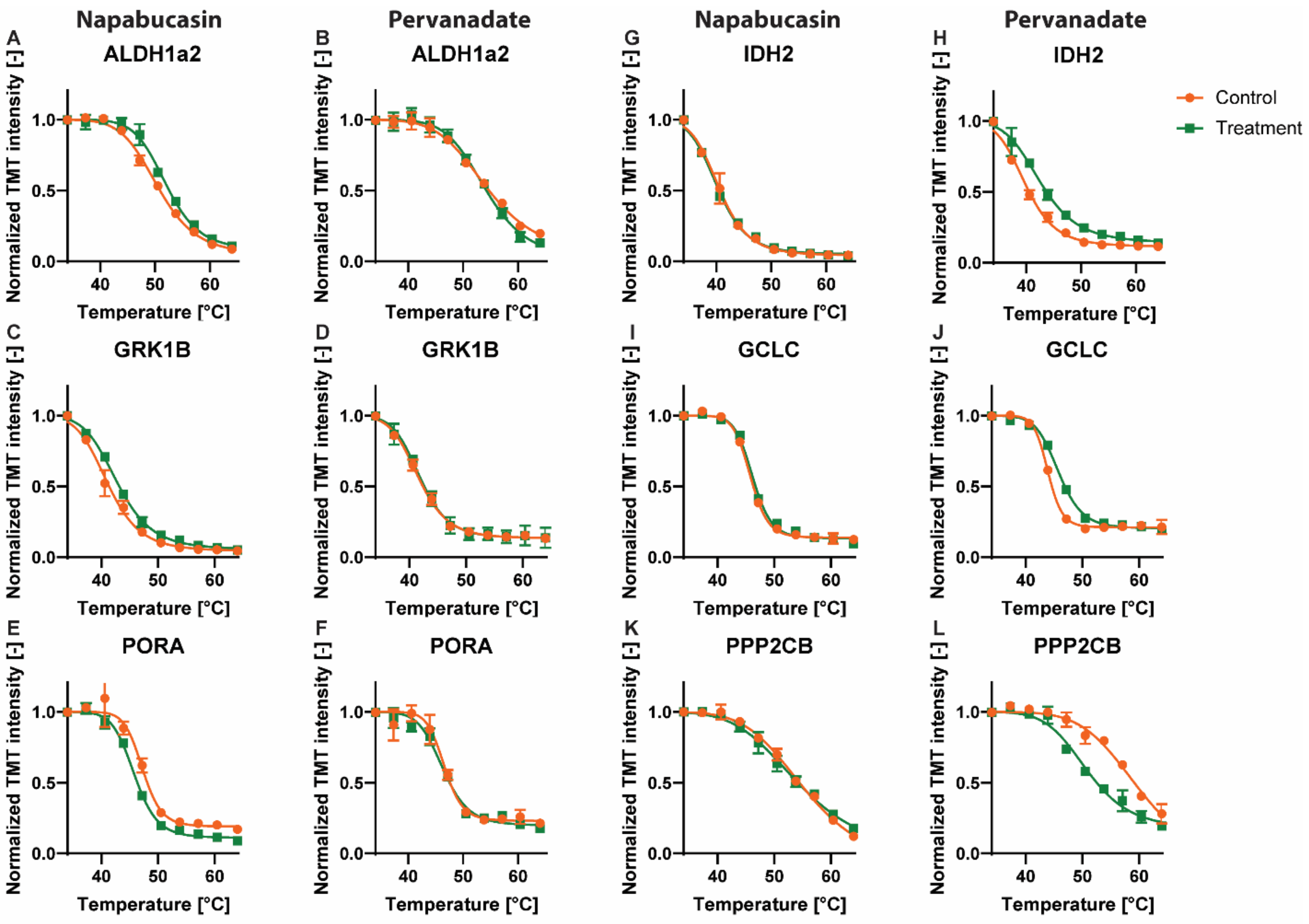

Supplementary Figure 3: Melting behavior of the same protein under different (Pervanadate or Napabucasin) treatment conditions. Melting curves for the proteins ALDH1a2, GRK1B, PORA, IDH2, GCLC and PPP2CB after Napabucasin or pervanadate treatment show that the induced shift is selective and unique for the treatment. Data points (n = 2 independent experiments) are shown as mean ± SEM, melting curve fitting was performed according to chemical denaturation theory.

A

Gene name	All peptides	Unique peptides	Unique sequence coverage [%]	Thermal shift?
**aldh1a2**	37	33	61.4	Yes
**aldh1a3**	15	12	30.4	Yes
**aldh1l1**	62	54	59	No
**aldh1l2**	62	54	63.1	No
**aldh2.1**	20	1	2.7	Yes
**aldh2.2**	28	9	17.6	No
**aldh3a2a**	9	8	18	Yes
**aldh3a2b**	24	23	45.9	No
**aldh3b1**	12	1	1.7	No
**aldh4a1**	20	20	33.3	No
**aldh5a1**	21	20	53.5	Yes
**aldh6a1**	23	23	57.1	No
**aldh7a1**	37	3	5.3	No
**aldh8a1**	19	19	45.6	No
**aldh9a1a**	44	42	77	No
**aldh16a1**	20	2	2.9	No
**aldh18a1**	24	24	37.7	No

B

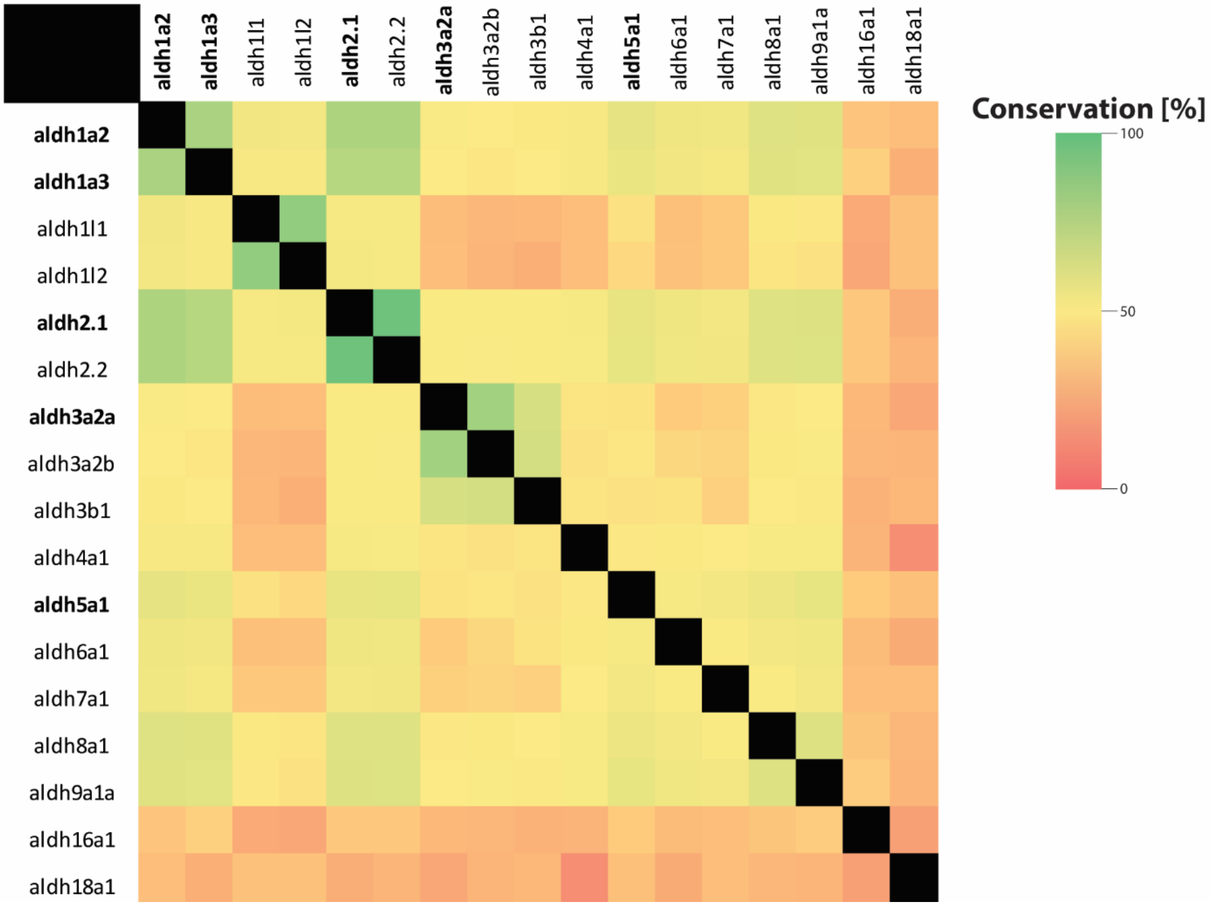

Supplementary Figure 4: Number of peptides identified and sequence coverage of all Aldh proteins found in the thermal proteome profiling experiment using napabucasin and whether they show a thermal stabilization effect (A). The heat map shows the conservation (in percentage) between all Aldh proteins found in the experiment (B). The pair wise conservation was determined using the EMBOSS Needle algorithm(1).

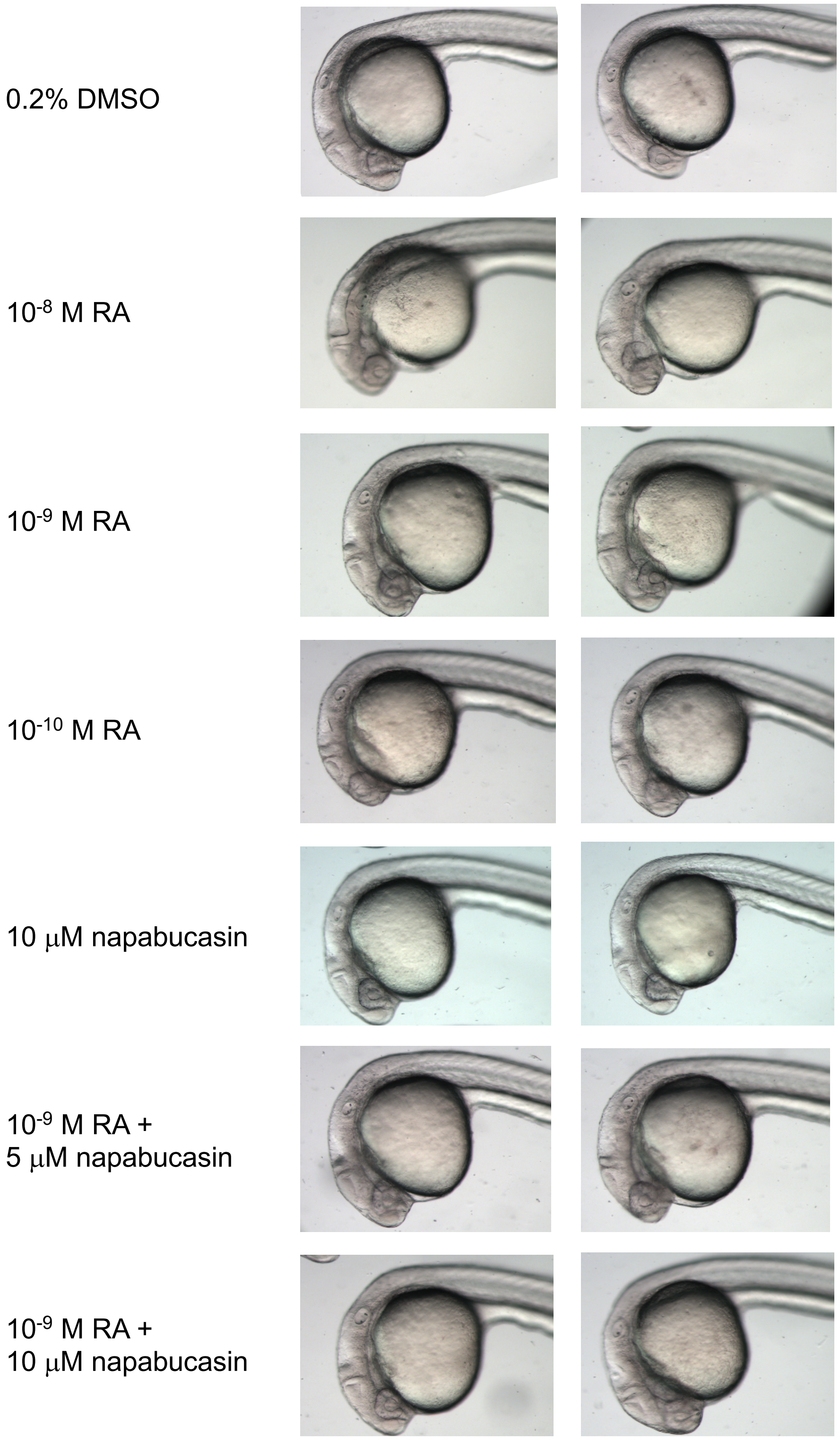

Supplementary Figure 5. Representative images of 28 hpf embryos that were used for measurement of the distance from the tip of the nose to the otolith (Fig. 4 A,B). Embryos were treated with the agents as indicated. Two representative embryos of each treatment are depicted, illustrating that the treatments did not induce gross developmental defects, but rather subtle defects which were quantified in Fig. 4B.

References

1. F. Madeira, *et al.*, The EMBL-EBI search and sequence analysis tools APIs in 2019. *Nucleic Acids Res.* **47**, W636–W641 (2019).
